# A Storytelling Approach on Vocabulary, Reading, and Letter Sound Fluency of Struggling First Graders With German as Second Language With and Without Behavioral Problems

**DOI:** 10.3389/fpsyg.2021.683873

**Published:** 2021-07-14

**Authors:** Anne Barwasser, Janine Bracht, Matthias Grünke

**Affiliations:** Department of Special Education and Rehabilitation, University of Cologne, Cologne, Germany

**Keywords:** storytelling, vocabulary, reading and letter sound fluency, German second language, behavior problems

## Abstract

The number of students learning German as a second language (L2) is steadily increasing. Unfortunately, studies reveal that less-proficient school performance affects a larger proportion of these students and additional behavioral problems can create even greater learning barriers. In order to master a language, the focus is not only on vocabulary, but also on reading, and studies show that multi-component intervention in reading and L2 acquisition is particularly promising. Therefore, this multiple baseline study focuses on a multi-component storytelling intervention on vocabulary, reading, and letter sound fluency of low-achieving first graders with German as L2 with and without behavioral problems (*N* = 7). The intervention was implemented 3 times a week over a 6-week period. Results show significant large to very large effects on vocabulary and moderate to large effects on letter sound fluency and reading, providing indication for the positive impact of storytelling on multiple aspects simultaneously for the focused sample.

## Introduction

### German as a Second Language

Education is largely dependent on language and in the German education system, the understanding and speaking of German at native language level is assumed (Becker-Mrotzek et al., 2012). According to the Federal Statistical Office, about 11% of the students at educational institutions have a migration background (Federal Statistical Office, 2020) and learning German as second language (L2; Aschenbrenner et al., 2016). The Programme for International Student Assessment (PISA) studies shows that students with a migration background perform significantly worse at school than students who learn German as their mother tongue [Organisation for Economic Cooperation and Development (OECD), 2019], and it has been shown that a large proportion of fourth graders do not or only partly speak German at home (Hußmann et al., 2017). German L2 students struggle in schools leading to a challenge for the teachers in designing appropriate lessons (Becker-Mrotzek et al., 2012) and a challenge for the students themselves with respect to educational opportunities.

### Hurdles for Second Language Learners

To be proficient in language, various skills within language acquisition, such as phonetics and literacy, are needed (Aschenbrenner et al., 2016). But especially vocabulary learning is immensely important (Schmitt et al., 2011), and it is shown that particularly students with L2 experience severe failure in this area (Webb and Chang, 2012). In addition, letter sound fluency (LSF) is essential for language communication and acquiring the sound of individual letters presents a particular hurdle (Kim and Piper, 2019), and students who struggle with LSF are more likely to have difficulty in their reading skills later on as well (Piasta and Wagner, 2010). A reason for this might be that children fail to read because their overall L2 competence is not yet sufficient to read adequately (Wallace, 2014).

Also, it is widely known that a certain struggle in language development, as vocabulary, expression, and reading, can be associated with problems in behavior (Peterson et al., 2013; Jansen et al., 2020). It has been reported that young children with language difficulties might develop problem behavior (Henrichs et al., 2013) which can get worse over time (Curtis et al., 2018). More specifically, deficits in language are connected to deficits in attention processing (Peterson et al., 2013) which can be linked to learning-related behavior (LRB). LRB, according to McClelland et al. (2006), includes abilities like staying focused, organizing school material, and working on one’s own. A meta-analysis by Chow and Wehby (2018) revealed a negative relationship between language deficits and problem behavior independent of age and time.

### Important Language Components

Vocabulary is fundamental but challenging in a L2 language and influences all stages of acquisition (Ender, 2016). Vocabulary can be differentiated between expressive and receptive. Receptive vocabulary is words which can be recognized but not actively spoken, whereas expressive vocabulary can be directly used (Schmitt, 2014). Significant correlations have been shown to exist between expressive vocabulary and reading ability in children from primary school (Wise et al., 2007). In general, it has been found that L2 vocabulary knowledge is linked to L2 reading comprehension (Lervåg and Aukrust, 2010). As in an L1, the automation of lower hierarchy processes, such as word recognition, is fundamental for comprehension (Kramer and McLean, 2019). The Dual-Route Model (DRM; Coltheart, 2005) describes two routes, the lexical and the non-lexical route, to show how readers read aloud. The lexical route refers to the mental lexicon where words can be automatically stored and retrieved [more important for irregular words: e.g., *“hoch” (high)* than for regular words: e.g., *“Sand” (sand)*]. The non-lexical route goes through the grapheme-phoneme correspondence (e.g., important for non-words like “*brelo*” or “*blustof*”). In terms of direct word recognition, the direct route is important, where sight words can be retrieved. Sight words are words that can be retrieved within 1 s of occurrence (Ehri, 2005). In addition to memorizing familiar words, letter sound knowledge (LSK) also plays an important role in the non-lexical route of DRM because it enables readers to decode unfamiliar words (Ehri, 2002). Both approaches should be possible for a reader to build up adequate reading competence in a language. Clemens et al. (2017) found that LSF, a sub-component, was predictive of subsequent reading fluency with respect to kindergarten children. Through a mediation analysis of results from a large-scale intervention study (*N* = 152), Hulme et al. (2012) showed that problems in LSK and phoneme awareness can cause difficulties in later word-reading-proficiency in 5-year-old children.

### Fostering Second Language Acquisition

In order to counteract hurdles in second language acquisition and to offer L2 students an opportunity to acquire an L2 adequately, it is necessary to provide effective support. The dual-coding theory (DCT; Paivio, 2008) states that there is a verbal way and a non-verbal way (i.e., pictures) to store information underlining the importance of presenting new input verbally and non-verbally in a language, especially for L2 students (Huang et al., 2019). The verbal way is related to linguistic information (e.g., sound) and the non-verbal system is linked to visual information (e.g., pictures; Paivio, 2007). According to Reed (2010) using both systems, maximizes the likelihood that information will be stored adequately.

Another way to train new content is either through explicit (intentional) training or implicit (incidental) training (Jin and Webb, 2020) – or a combination of both (Choo et al., 2012). Intentional learning means that the learner is aware of learning something, and incidental learning means that the learner learns something like a by-product without being aware of it (Webb and Nation, 2017). In the case of incidental learning, it has been said that words are easier to acquire through repeated occurrence in context (Webb and Nation, 2017). Marulis and Neuman (2010) conducted a meta-analysis about the impact of vocabulary interventions on the language development of pre-K and kindergarten children and found an overall effect size of *g =* 0.88 of vocabulary training on word learning. Moreover, it was found that a combination of implicit training and explicit training lead to a higher effect size (*g =* 1.21) than explicit (*g =* 1.11) and implicit (*g =* 0.62) in isolation. Hulme et al. (2012) found that teaching LSK and phoneme awareness explicitly in a reading and phonology intervention lead to an improvement of these two abilities.

It has been shown that multi-component supports, including among other, phonics, vocabulary, and fluency increases the probability of becoming a good reader (Foorman and Torgesen, 2001). A recently published literature review by Donegan and Wanzek (2021) showed that multi-component reading interventions for elementary school with the highest effects incorporate instruction in decoding on the word level and in summary multi-component interventions are promising with regard to improve foundational reading skills and reading comprehension.

### Storytelling

Listening to stories has been known to influence language development on different areas positively in children (Isbell et al., 2004). Storytelling is a procedure where a teller tells a story in an authentic environment using gestures, vocalization, and images to convey a certain message to the audience who are incorporated in the storytelling procedure (Mello, 2001). Storytelling has the ability to engage learners personally (Brewster et al., 2002), motivate learners, and spark interest in the subject matter (Wright, 2013). Using storytelling does have positive impacts on child’s oral and written language development (Fien et al., 2011 ; Baker et al., 2013) and through the procedure of storytelling facts as well as vocabulary can be memorized better (Wajnryb, 2003). Lenhart et al. (2018) focused on the impact of story listening on vocabulary acquisition and found that vocabulary was acquired incidentally without any word explanation with a moderate effect (*d* = 0.37) which was in turn not stable over time (age 3–6) concluding that using only incidental vocabulary training might not be sufficient enough. A meta-analysis by Mello (2001) indicates that using storytelling led to gains in vocabulary, fluency, and writing skills, among other variables. Suggate et al. (2013) examined storytelling in second and fourth grade German readers and revealed that more freely storytelling has more benefits than simply reading the story.

Read aloud has been shown to be effective for vocabulary, comprehension, and narrative language in first graders (Baker et al., 2020) and for phonological awareness (Swanson et al., 2011). Since storytelling belongs rather to the implicit method, adding flashcards to storytelling in order to teach components explicitly would be, according to Marulis and Neuman (2010), a further boost in effectiveness. Two additional studies by Barwasser et al. (2020) and Knaak et al. (2021) investigated a combined storytelling intervention consisting of implicit and explicit components on vocabulary acquisition in English language learning of students with and without learning disabilities showing that this combination is effective in the context of vocabulary acquisition. Barwasser et al. (2021) went a step further and examined the combined storytelling method in German second language learners from primary school on vocabulary and reading with overall positive effects.

### Motivation and Self-Graphing

In second and foreign language acquisition, the ability to increase competence in a language often depends on how motivated a learner is (Ghenghesh, 2010). Adding motivational components to an intervention can be specifically successful (Bowman-Perrott et al., 2013; Leko, 2016). It has been shown that incorporating self-monitoring procedures, such as self-graphing, the visualization of a student’s own progress showing earlier scores and current scores (Stotz et al., 2008; Guzman et al., 2018; McKenna and Bettini, 2018), reading achievement can be improved for students with disabilities (Laurice and Eveleigh, 2011) and on task behavior as well as general academic productivity (DiGangi et al., 1991). Self-graphing can be realized by providing students with a graph overview where they can enter their scores after each measurement point in order to follow their own learning progress step by step. A meta-analysis by Guzman et al. (2018) revealed large effects of self-monitoring procedures on reading performance in K-12 students (*TauU* = 0.79, *p <* 0.001).

### Research Questions

Based on the knowledge that there is a large number of low-performing German as a L2 language students in Germany, with both behavioral problems and motivation playing a significant role, a multi-component storytelling intervention was designed to simultaneously address three important components in language learning: vocabulary, LSF, and sight word reading, and to investigate its effects on German L2 students with and without behavioral problems. In addition, we have implemented a social validity questionnaire in order to figure out the acceptance of the intervention rated by the participants. Assessing social validity is a necessity to crystallize the acceptance and usefulness of interventions (e.g., Briesch et al., 2013). Accordingly, the four research questions are as follows:

1. Does a multi-component storytelling intervention lead to an increase in expressive vocabulary in German L2 students with and without behavior problems?
2. Does a multi-component storytelling intervention lead to an increase in LSF in German L2 students with and without behavior problems?
3. Does a multi-component storytelling intervention lead to an increase in sight word reading in German L2 students with and without behavior problems?
4. How was the intervention evaluated by the participants in terms of social validity?

## Materials and Methods

### Participants and Setting

The present study was conducted at an inclusive elementary school in a large city in North Rhine-Westphalia, Germany, targeting grade 1. To participate in the study, teachers of the respective classes were to identify all students who met the criterion “German as a second language” (*N* = 10). In addition, appropriate parental consent to participate in the study had to be obtained. The teachers received a teacher questionnaire to obtain relevant information on the proposed students regarding socio-demographic characteristics (see Table 1).

**Table 1:** Characteristics of the participants.

	Lio	Kim	Tila	Nele	Niek	Abden	Elif
Age	6;3	6;5	7;1	6;6	6;3	7;1	6;2
Grade	1	1	1	1	1	1	1
Gender	Male	Female	Female	Female	Male	Male	Female
L1	Polish	Polish	Turkish	Chinese	Italian	Turkish	Turkish
LRB	12	4	13	3	14	10	6
Reading W (PR)	<7	<7	<2	9–13	7–8	16–17	5–11
Reading P (PR)	<2	<2	<4	24	6–8	19–23	8–10
**BAKO (PR)**							
Subtest PS	2	2	2	2	48	2	21
Subtest VS	42	6	19	53	6	6	61
Subtest RD	3	3	3	21	3	3	34
Subtest PI	8	8	8	74	8	8	21
Subtest SC	28	15	42	71	1	28	28
Subtest VD	57	7	23	57	23	23	57
Subtest WR	9	9	9	35	23	9	35
Total	7	0	2	39	2	1	31
Vocab (PR)	12	5	15	26	21	27	16

PR, percentile; W, words; P, pseudowords; LRB, learning-related behavior (cutoff 10); L1, native language; PS, pseudoword segmentation; VS, vowel substitution; RD, residual word determination; PI, phoneme interchange; SC, sound categorization; VD, vowel length determination; WR, word reversal; and Vocab, German vocabulary test.

#### German Vocabulary Test

A vocabulary test (WS/ZF-R; Weiß, 2007) in the form of a group screening was used first to assess the students’ verbal language skills. The WS/ZF-R measures colloquial vocabulary beyond the basic vocabulary of the German language and is used to determine the developmental level of verbal skills of students. The test sheet contains 30 multiple-choice items with five alternative answers each. Each task consists of a key word being given first. Subsequently, the respondents have to select the word from the five alternative answers that has a similar meaning as the given keyword. The reliability of the WS/ZF-R was assessed using the split-half method (*N* = 618), where values ranged from rtt = 0.79 to rtt = 0.90 with a mean value of rtt = 0.87. For the correlation with German grades (*N* = 689), the value was *r* = 0.48 (Weiß, 2007). The results are shown in percentiles (PR) and a PR under 15 means underdeveloped. For example, a percentile of 15 means 15 percent of the subjects in the norm sample scored the same or fewer points. The participant with a PR of 15 therefore belongs to the 15 percent of the weakest in his age group.

#### SLRT II

The Salzburg Reading and Spelling Test (SLRT II; Moll and Landerl, 2010) was used to assess reading ability at the word and pseudoword level. These two subtests each consisted of a one-minute reading fluency test by reading given words and pseudowords. The total time required is time-efficient at approximately five minutes. The parallel test reliability ranges from 0.90 to 0.98 and correlations with other German reading tests range from 0.69 to 0.92. All participants who had a percentile below 15 were selected for the study.

### Test for Phonological Awareness (BAKO 1–4)

A test for phonological awareness for grades 1–4 was additionally used (BAKO 1–4; Stock et al., 2017). There are a total of 174 tasks divided into seven subtests: (1) pseudoword segmentation, (2) vowel substitution, (3) residual word determination, (4) phoneme interchange, (5) sound categorization, (6) vowel length determination, and (7) word reversal. The time required to complete the test is approximately 30 min. Norms are available for each grade level (*N* = 876) and reliability shows that internal consistently varies by grade level (between *α* = 0.90 and *α* = 0.92, split-half reliability between *r* = 0.90 and *r* = 0.94). Criterion-related validity with reading or spelling performance measured by standardized tests varies by grade level between *r* = 0.42 and *r* = 0.68 (Stock et al., 2017). Results are again shown in PR.

### Integrated Teacher Report Form

The integrated teacher report form (ITRF; Volpe et al., 2018) represents a multilevel screening procedure used to identify student behavior difficulties. In the present study, the ITRF-G short version is applied, which is the German translation of the English version. In the research conducted, the screening is conducted by the classroom teachers as they are in the best position to assess the students’ behavior. The teachers assess specific behaviors of the students on an assessment sheet, and the items are created based on the factors “learning-related behavior” and “oppositional/disruptive behavior.” The ITRF-G is administered in a short version with 16 items, whereas the original version includes 47 items. The conducted short version has been positively evaluated and shows high internal consistency and sufficient test-retest reliability in terms of reliability and high external validity for all scales in terms of validity. The cutoff value for learning-related behavior is 10 showing problems in this area (Volpe et al., 2018).

### Word Pretesting

To crystallize the final training words and to ensure that the words were not stored in either the expressive vocabulary or the mental lexicon for reading, words were auditioned prior to the study. Once for expressive vocabulary and once for reading. The pool of words (*N* = 143) came from the Metacom symbols (Kitzinger, 2020) and care was taken to ensure that words were taken which the children could use well in everyday life. These words were queried both expressively and in reading. For the reading test (day 1), the 143 words were integrated into a powerpoint presentation so that one word was on each slide individually. The slides were scrolled in 1-s intervals, since according to Ehri (2005), a word is considered a sight word if it can be read within 1 s of its occurrence. Here, all words that could not be read were marked.

After a few days (day 2), the expressive test was performed with the exact same words. Here, the children were not shown the 143 words, but pictures matching the words. Here, too, there was a picture on a slide – there was no time limit. Now, for each picture, the children were asked what the word was called. All non-conscious words were marked and compared with the reading words. A total of 40 word-overlaps resulted for unknown expressive words and words not read correctly. The 40 training words in reading were the same as in vocabulary for the intervention and measurements later on. Thus, the children could neither read these words nor express them actively. The 40 training words, which were selected together with the teachers, had a mid-frequency of *M* = 10.5, meaning that the words appear 10.5 times per million words in a corpus (Brysbaert et al., 2018). To estimate the frequency, we used the childLex database (Schroeder et al., 2015).

The students (*N* = 10) are divided into three groups. Group 1 had three children, group 2 had three children, and group 3 had four children. All participants learned German with the entry of kindergarten at age 3–3; 5. According to COVID-19 rules, groups were not allowed to be mixed across classrooms. Each group has a different baseline time and thus starts the intervention with a time delay. Three children are dropped from the data because they have too much missing data due to COVID-19 quarantine regulations. As a result, the finale sample for this paper is *N* = 7.

### Design

The present research utilized a multiple baseline design across participants to examine the effects of the intervention. A single case analysis is often understood to be a study of one individual. However, a multiple baseline design embeds subcases within an overall case. The introduction of the intervention is temporally staggered across the subjects. The goal of implementing a multiple baseline design is to substantiate a cause-effects relationship by demonstrating that changes in the dependent variable only occur when the treatment is given (Lane et al., 2017). First, a baseline of varying length is performed with 5–7 sessions. After each of these sessions, the dependent variables were collected. After completion of the baseline phase, the intervention starts in the following sessions. Data were also collected after each intervention session (e.g., baseline 1 – measurements; baseline 2 – measurements – … intervention 1 – measurements; intervention 2 – measurements; and intervention 3 – measurements). Each group was randomly assigned to a specific baseline length resulting in group 1 = 5 baselines, group 2 = 6 baselines, and group 3 = 7 baselines. The baseline and intervention sessions took place 3 times a week for 25 min, after which the children were measured individually for each of the three dependent variables. The entire period spanned 6 weeks and 1 week of diagnostic testing. Due to a previous school closure because of COVID-19, the study started later and comes to 18 measurement time points of originally planned 24. Two master’s students for special needs education functioned as test leaders and interventionists. Both supported each group together.

### Dependent Variables and Measurement

In total, there are three dependent variables: expressive vocabulary, sight word reading, and LSF. The 40 training words were used for expressive vocabulary and reading. For LSF, all letters from the German alphabet were measured.

1. **Expressive vocabulary**: The 40 training words were packed into a powerpoint presentation in the form of pictures, with one picture per slide. For each picture, the child was asked if he knew the name of the word. The total number of correctly conscious words expressive was transferred to an excel table per measurement point.
2. **LSF:** All letters of the German alphabet were mixed and written on two 8.3 × 11.7-inch sheets, so that a total of 104 letters could be seen. The child was now asked to pronounce as many sounds as possible correctly within 1 min. A timer was set to 1 min and the two test leaders listened attentively. The total number of correctly pronounced sounds was also entered in the excel table for each measurement point.
3. **Reading:** The 40 training words written were embedded in a powerpoint. Here, one word per slide was written down. The slides were separated by hashtags and were laid out in 1-s intervals (see Ehri, 2005). Again, the total number of correctly read words was recorded in an excel table per measurement time point.

### Intervention Material

**Figure 1:**
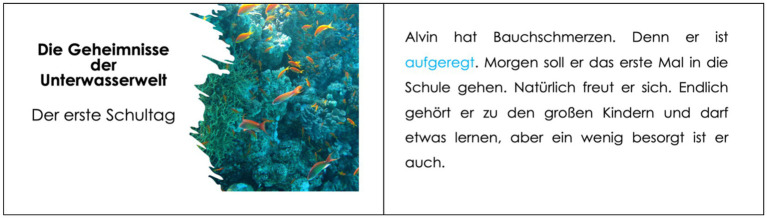
Example part of a story. The secrets of the underwater world. The first day of school. Text: Alvin has a stomach-ache because he is excited. Tomorrow he is supposed to go to school for the first time. Of course, he is looking forward to it. Finally, he belongs to the big kids and is allowed to learn something, but he is also a little worried.

For the direct instruction of the words and the sounds, a phonetic table and 8.3 × 11.7-inch flashcards with the letters on them and 8.3 × 11.7-inch flashcards with the picture and the matching word were used. For the storytelling intervention, short stories were required for each session. Before the study started, the master students talked to the children about their interests in order to determine the focus of the stories. In total, there was one full story with sub-chapters per session. The stories (example Figure 1) were self-written with somewhat the same length and formatting. Additionally, care was taken to ensure that all words occurring were not too difficult. The training words were always embedded and from the pool of 40 words always five were taken into one story which appeared twice on one story. The words were randomly assigned to the stories, making sure that in the end the words occurred in equal proportions. The training words in the story were always highlighted in blue, while the rest of the font was black.

**Figure 2:**
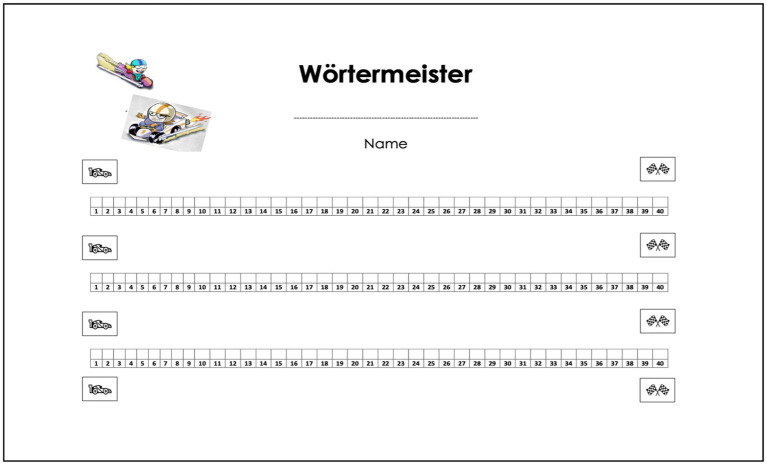
Example of self-graphing sheet. Wörtermeister = word master.

Regarding the motivational system, there were three self-graphing sheets for the children corresponding to the three dependent variables. Each sheet consisted of several rows one below the other, which were supposed to represent the sessions (example Figure 2). The rows consisted of small boxes that were supposed to represent the number of words/sounds correctly known where the participants were asked to color the amount of correct known words/sounds after each measurement point.

### Procedures

#### Baseline

The baseline (A phase) is used to record the actual state in a multiple baseline design. Before the storytelling intervention starts, all three groups go through a baseline phase of different lengths for the groups. The baseline activities must not have anything to do with reading, vocabulary, or LSF, so that the dependent variables are not already promoted in the baseline. Thus, during baseline condition, games, puzzles, and math problems are solved together in 25 min. These are simple tasks that do not explicitly promote vocabulary, reading, or the LSF. Afterward, the three dependent variables are measured for each child.

#### Storytelling

After the baseline (A) phases are all completed, the groups begin the intervention phase (B phase). The group constellations remain unchanged. Storytelling can be divided into two stages. In the first stage (10 min), the kids sit in a semi-circle around the interventionist who is firstly introducing the words to be learned directly to the participants. Both, the words and letters of the last story (despite session one), are repeated, and the words and letters of the current story are introduced through flashcards and a phonics table. In order not to overtax the children, only 10 of the 40 words are directly instructed per session. The interventionist holds up the flashcard with the word and the picture, covers the written word, and asks the children, based on the picture, whether they know what it means. Then, they talk about the word. Next, the interventionist uncovers the written word and asks the children if anyone can read the word aloud. Subsequently, everyone reads together and then, the interventionist reads the word again. After that, the interventionist lifts up the phonics picture. For each intervention session, 10 sounds were randomly selected to be trained. Using the phonics picture and the words, the interventionist asks, for example, for an “L”: “Who knows how to pronounce that?” “And can you find the sound in one of our words?” The procedure lasts 10 min.

The second stage (15 min) involves the process of storytelling. The stories were learned by heart by the interventionists and the text serves the children to follow the story and see the marked training words. Each story is told out loud to the students and if a training word is appearing in the story, the story is paused and the word, as well as one sound, is discussed using the appropriate flashcards (a word with a matching picture). After the storytelling, the three measurements are carried out with each child individually and feedback on the learning process follows on the self-graphing sheets. Each time after the measurement, each child enters the number of correct known items in two separate self-graphing sheets for the amount of correctly read words and correctly known word expressively.

#### Treatment Fidelity

In order to record treatment fidelity in the present study, the experimenters were first provided with a detailed script with steps to be followed. Additionally, the implementers were given a checklist to complete at the end of each intervention session without being aware of the intention of the sheet. This was used to reflect on compliance with what was outlined in the script. The checklist is divided into six sections: environment/external circumstances, planning, materials, procedure of support, diagnostics/feedback, and handling student behavior during support using three response options (“yes”; “no”; “not applicable”). In addition, a free field was available to the investigators for comments on special features in the context of the support. The inter-rater reliability is 100%.

#### Social Validity

To measure the acceptance of the intervention by the students, a questionnaire was designed within the framework of social validity, which was handed out to the students at the end of the support. The interventionists were not present in order to avoid biased results and to obtain an honest opinion from the students. The questionnaire contains nine items which should be rated on a scale from 0 (= completely not agree) to 4 (= completely agree). The items were as follows: *(1)* *Storytelling helped me to be able to read words correctly*; *(2)* *Storytelling helped me learn words and their meanings*; *(3)* *Storytelling helped me to pronounce sounds correctly*; *(4)* *I understood well the meaning of the promotion*; *(5)* *I have learned a lot during storytelling*; *(6)* *I gladly came to the intervention sessions*; *(7)* *The self-graphing sheets were fun*; *(8)* *The stories were great*; and *(9)* *I would like to do more with stories in school*.

#### Data Analysis

**Figure 3:**
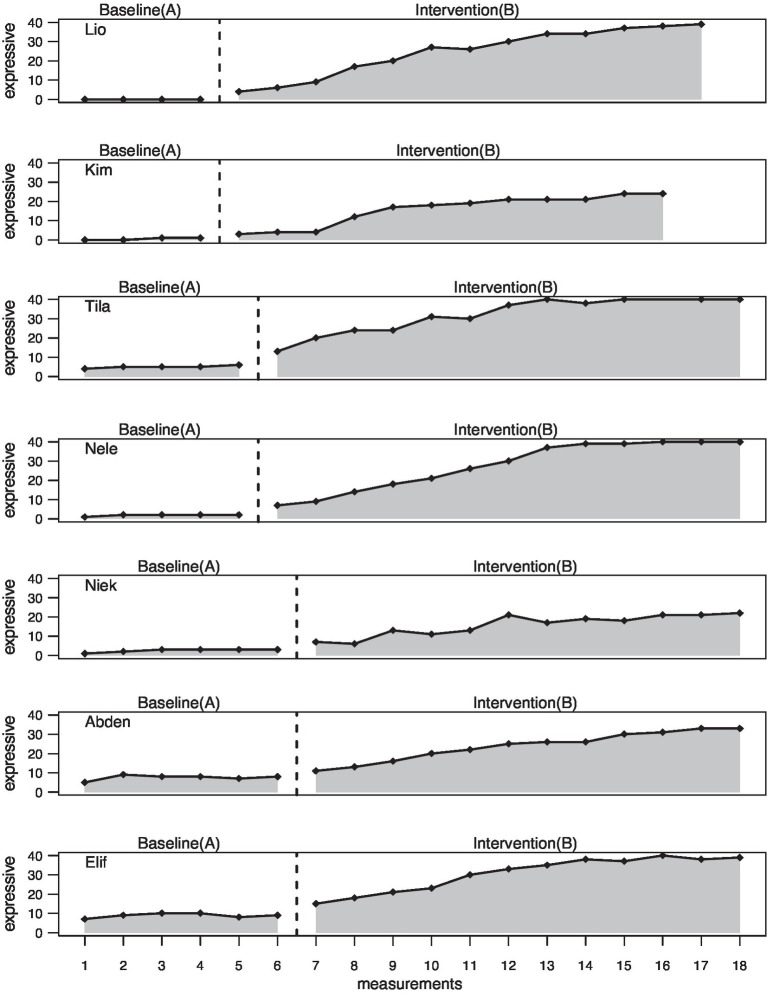
Amount of known expressive vocabulary.

**Figure 4:**
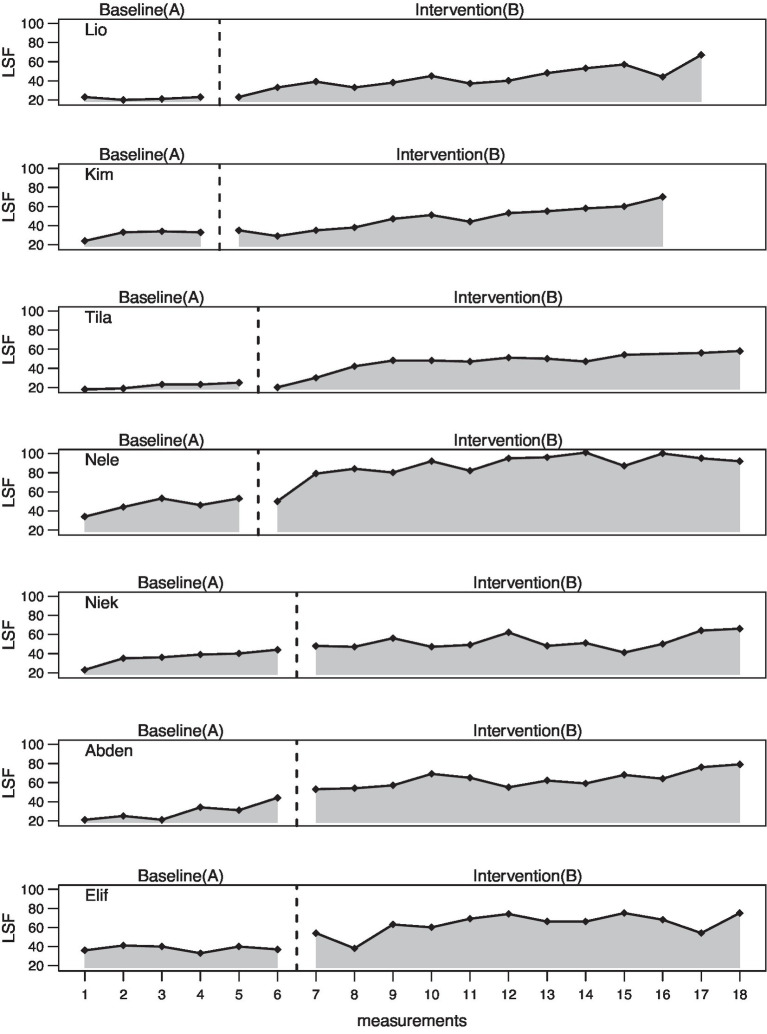
Letter sound fluency (LSF) in 1 min.

**Figure 5:**
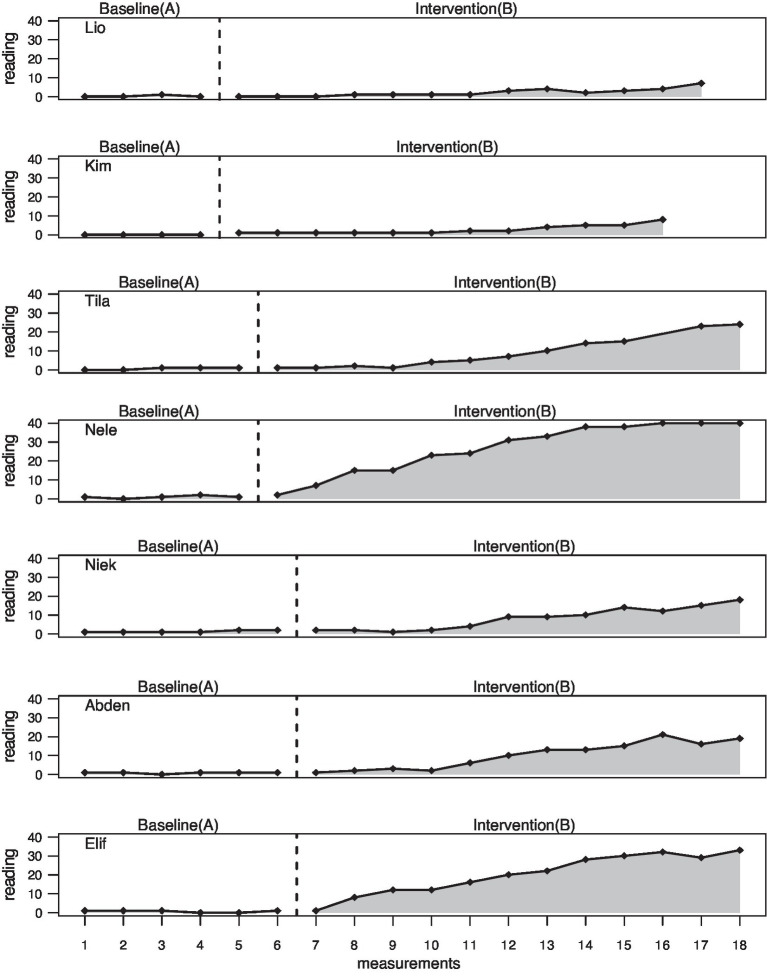
Amount of correctly read sight words.

The entire data analysis was done using the statistics program “R” and the Scan Package for multiple baseline design analysis in order to estimate the intervention (B phase) effects compared to the baseline (A phase). The graphs (Figures 3–5) for each dependent variable serve for visual analysis. In addition, mean and median values of the two phases as well as the maximum values in phase A and phase B were determined and mean baseline difference (MBDi). MBDi is a non-parametrical method which measures increase of a certain output from baseline (O’Brien and Repp, 1990). Further, overlap measures were used including the non-overlap of all pairs (NAP, Parker et al., 2011a), the percentage exceeding the median (PEM, Ma, 2006), the percentage of all non-overlapping data (PAND; Parker et al., 2007), and finally, the Tau-U additionally considering an A phase trend using the formula: A vs. B + TrendB − TrendA. TauU measures data non-overlap between phase A and phase B (Parker et al., 2011b).

The single-case reporting guidelines by Tate et al. (2016) suggest the use of inferential statistics to directly test for treatment effects. Even though there is still no universal gold standard for analyzing data from respective experiments, hierarchical piecewise regression modeling has become the most common tool for investigating the null hypothesis (Raudenbush and Bryk, 2002; Manolov et al., 2010; Waddell et al., 2011). In this approach, the data points during baseline of one individual are used to calculate a regression line and estimate the progression of the data during the intervention. Changes in level and/or slope across phases can then be tested for statistical significance (level 1 analysis). Subsequently, data over several individuals can be accumulated to examine causal elements behind treatment effectiveness (level 2 analysis). When regression modeling is used in group studies, each data point stems from a different individual. However, if this approach is applied in single case level 1 research, the data points stem from one and the same person. One of the basic requirements for using parametric statistics (like regression analysis) is the independency of the distributed errors. There is no logical reason to assume that errors of different individuals are statistically associated. In contrast, the danger of autocorrelation in single case research is ever present. For example, it is anything but unlikely that errors in observations that are close together in time are more similar than those that are more distant. The degree to which they correlate corresponds with the risk of incorrectly rejecting a true null hypothesis. To reduce the likelihood of mistakenly dismissing the absence of a given effect, we used a statistical package for R called SCAN (Wilbert, 2021) that controls for autocorrelation in single case data.

## Results

### Expressive Vocabulary

Overall, the visual baseline is very flat for all participants and there is a steady increase in the B phase. Tila (*M* = 5.00), Abden (*M* = 7.50), and Elif (*M* = 8.83) start with slightly higher values in the A phase while Lio (*M* = 0.00), Kim (*M* = 0.50), Nele (*M* = 1.80), and Niek (*M* = 2.50) start very low (see Table 2). The highest mean value in the B phase is shown by Tila (*M* = 31.42), and the lowest value is found in Niek (*M* = 15.75). The highest increase is shown by Kim (3,034%) and Lio (2,469%), and the lowest increase is shown by Abden (217.73%) and Elif (246.32%). Tila, Nele, and Elif reach the maximum possible score of 40.00 in the B phase (see Figure 3).

**Table 2:** Descriptive data for expressive vocabulary.

Participants	N(A)	N(B)	M(A) SD	M(B) SD	MBDi	Md A	Md B	Max A	Max B
Lio	4	14	0.00 (0.00)	24.69 (12.41)	2,469%	0.00	27.00	0.00	39.00
Kim	4	14	0.50 (0.58)	15.67 (7.91)	3,034%	0.50	18.50	1.00	24.00
Tila	5	13	5.00 (0.71)	31.42 (9.30)	528,4%	5.00	34.00	6.00	40.00
Nele	5	13	1.80 (0.45)	27.69 (12.61)	1,438,4%	2.00	30.00	2.00	40.00
Niek	6	12	2.50 (0.84)	15.75 (5.63)	527,6%	3.00	17.50	3.00	22.00
Abden	6	12	7.50 (1.38)	23.83 (7.57)	217,73%	8.00	25.50	9.00	33.00
Elif	6	12	8.83 (1.17)	30.58 (8.98)	246,32%	9.00	34.00	10.00	40.00

N, measurements; M, mean; SD, standard deviation; MBDi, mean baseline difference; Md, median; Max, maximum; A, A phase; and B, B phase.

With regard to the overlap measures, the NAP shows the maximum value of 100.00 across all subjects (*p <* 0.001 – *p* < 0.01). The same picture can be seen for the PEM and the PAND. The Tau-U also shows statistically significant values (*p <* 0.001) which can be interpreted as a large change for Kim (0.69), Tila (0.70), and Niek (0.74) and as a very large change for Lio (0.83), Elif (0.84), Nele (0.88), and Abden (0.89; see Table 3).

**Table 3:** Overlap indices for expressive vocabulary.

Participant	NAP	*p*	PEM	PAND	TauU	*p*
Lio	100.00	<0.01	100.00	100.00	0.83	<0.001
Kim	100.00	<0.01	100.00	100.00	0.69	<0.001
Tila	100.00	<0.001	100.00	100.00	0.70	<0.001
Nele	100.00	<0.001	100.00	100.00	0.88	<0.001
Niek	100.00	<0.001	100.00	100.00	0.74	<0.001
Abden	100.00	<0.001	100.00	100.00	0.89	<0.001
Elif	100.00	<0.001	100.00	100.00	0.84	<0.001

NAP, non-overlapping of all pairs; PEM, percentage of data points exceeding the median; and PAND, the percentage of all non-overlapping data.

The results of the regression analysis reveal for group 1 a statistically significant slope effect from A phase to B phase (*p <* 0.05) with a beta coefficient of 2.464 and thus, an improvement by this value per intervention session. Group 2 shows a statistically significant level effect (*p <* 0.01) as well as a slope effect (*p <* 0.01) with an improvement of 2.379 per session. For group 2, a significant level effect (*p <* 0.05) and slope effect (*p <* 0.001) can also be seen with a beta coefficient of 1.668. As expected, a statistically significant level effect (*p <* 0.01) from the A phase to the B phase and a significant slope effect (*p <* 0.001) from the A phase to the B phase can be seen. The subjects managed to improve by 2.259 more expressive correctly conscious words per intervention session (see Table 4).

**Table 4:** Regression model for expressive vocabulary across all participants (level 2-analysis).

	*B*	SE	*t*	*p*
*Group 1*				
Intercept	−0.250	3.805	−0.66	0.95
Trend	0.200	1.170	0.171	0.87
Level	1.697	2.693	0.630	0.53
Slope	2.464	1.188	2.075	<0.05
*Group 2*				
Intercept	2.500	3.005	0.832	0.41
Trend	0.300	0.791	0.379	0.71
Level	7.231	2.437	2.966	<0.01
Slope	2.379	0.814	2.924	<0.01
*Group 3*				
Intercept	5.311	3.326	1.597	0.12
Trend	0.276	0.405	0.681	0.50
Level	3.784	1.611	2.349	<0.05
Slope	1.668	0.429	3.883	<0.001
*Overall*				
Intercept	3.456	2.229	1.550	0.12
Trend	0.140	0.402	0.349	0.73
Level	4.086	1.369	2.985	<0.01
Slope	2.259	0.417	5.415	<0.001

### Letter Sound Fluency

Visually, it can be said that the baselines here are not so flat compared to the expressive vocabulary and that positive trends can be partially assumed. Lio (*M* = 21.75), Kim (*M* = 31.00), and Tila (*M* = 21.60) start relatively low and also show no trend tendency in the A phase (see Table 5). Nele (*M* = 46.00), Niek (*M* = 36.17), Abden (*M* = 29.33), and Elif (*M* = 37.83) start with slightly higher values and show a positive trend tendency. Overall, however, there is also a clear increase for each test person in the B phase (see Figure 4).

**Table 5:** Descriptive data for LSF.

Participants	N(A)	N(B)	M(A) SD	M(B) SD	MBDi	Md A	Md B	Max A	Max B
Lio	4	14	21.75 (1.50)	42.85 (11.50)	97,01%	22.00	40.00	23.00	67.00
Kim	4	14	31.00 (4.69)	47.92 (12.16)	54,58%	33.00	49.00	34.00	70.00
Tila	5	13	21.60 (2.97)	45.92 (10.90)	112,59%	23.00	48.00	25.00	58.00
Nele	5	13	46.00 (7.84)	87.50 (13.35)	90,22%	46.00	92.00	53.00	101.00
Niek	6	12	36.17 (7.19)	52.42 (7.81)	44,93%	37.50	49.50	44.00	66.00
Abden	6	12	29.33 (8.91)	63.41 (8.46)	116,20%	28.00	63.00	44.00	79.00
Elif	6	12	37.83 (3.06)	63.50 (10.79)	67,86%	38.50	66.00	41.00	75.00

N, measurements; M, mean; SD, standard deviation; MBDi, mean baseline difference (MBDi); Md, median; Max, maximum; A, A phase; and B, B phase.

The overlap measures showed strong effects (94.00–100.00) for all children in the NAP, which were also statistically significant (*p <* 0.01 – *p* < 0.001). The PEM shows a maximum value of 100.00 for Lio, Nele, Niek, and Abden and a value of 91.67 for Kim, Tila, and Elif. The PAND also shows that the intervention was highly effective for all subjects (91.18–100.00). The Tau-U, taking into account a possible A phase trend, shows a moderate effect for Niek (0.52; *p <* 0.01), and a large change for the remaining children (0.62–0.69; *p <* 0.001; see Table 6).

**Table 6:** Overlap indices for LSF.

Participant	NAP	*p*	PEM	PAND	TauU	*p*
Lio	98.00	<0.01	100.00	91.18	0.69	<0.001
Kim	94.00	<0.01	91.67	93.75	0.64	<0.001
Tila	95.00	<0.01	91.67	95.00	0.62	<0.001
Nele	97.00	<0.001	100.00	96.92	0.64	<0.001
Niek	99.00	<0.001	100.00	98.61	0.52	<0.01
Abden	100.00	<0.001	100.00	100.00	0.67	<0.001
Elif	96.00	<0.01	91.67	95.83	0.64	<0.001

NAP, non-overlapping of all pairs; PEM, percentage of data points exceeding the median; and PAND, the percentage of all non-overlapping data.

Regression analysis showed neither a significant level effect (p = 0.50) nor slope effect (p = 0.38) for group 1. The same can be said for group 2. Group 3, on the other hand, shows a statistically significant level effect from the A to the B phase (*p* < 0.05), but also a trend in the A phase (*p* < 0.05). Overall, there is a significant level effect (*p* < 0.05) and an A phase trend (*p* < 0.01; see Table 7).

**Table 7:** Regression model for LSF across all participants (level 2-analysis).

	*B*	SE	*t*	*p*
*Group 1*				
Intercept	22.750	4.911	4.633	<0.001
Trend	1.450	1.529	0.949	0.35
Level	−2.410	3.520	−0.685	0.50
Slope	1.401	1.552	0.902	0.38
*Group 2*				
Intercept	25.100	14.713	1.706	0.10
Trend	2.900	1.838	1.578	0.13
Level	10.240	5.663	1.808	0.08
Slope	−0.442	1.890	−0.234	0.82
*Group 3*				
Intercept	25.664	4.480	5.724	<0.001
Trend	2.514	1.068	2.354	<0.05
Level	9.532	4.244	2.246	<0.05
Slope	−1.050	1.131	−0.928	0.36
*Overall*				
Intercept	23.614	5.182	4.557	<0.001
Trend	2.666	0.807	3.304	<0.01
Level	5.668	2.742	2.067	<0.05
Slope	−0.470	0.838	−0.561	0.58

### Reading

Visual inspection shows enormously flat baselines with no positive trends. Significant increases in the B phases can only be found for five children. Lio and Kim initially reveal no improvement until the end, when there is a discrete increase. Kim (*M* = 0.00) and Lio (*M* = 0.25) start with the lowest values in the A phase and Niek (*M* = 1.33) and Abden (*M* = 0.83) with the highest values. The highest mean values in the B phase are shown by Nele (*M* = 26.62) and Elif (*M* = 20.25) and the lowest values by Lio (*M* = 2.08) and Kim (*M* = 2.67; see Table 8). The largest increase from A to B phase is observed in Nele (2562%) and Elif (2922%), and the least increase can be seen in Kim (267%) and Lio (732%). Only Nele reaches the maximum value of 40.00 in the B phase (see Figure 5). Lio and Kim show the lowest values with a maximum of 7.00–8.00.

**Table 8:** Descriptive data for words read correctly.

Participants	N(A)	N(B)	M(A) SD	M(B) SD	MBDi	Md A	Md B	Max A	Max B
Lio	4	14	0.25 (0.50)	2.08 (2.06)	732%	0.00	1.00	1.00	7.00
Kim	4	14	0.00 (0.00)	2.67 (2.31)	267%	0.00	1.50	0.00	8.00
Tila	5	13	0.60 (0.55)	8.92 (8.36)	1,386,67%	1.00	6.00	1.00	24.00
Nele	5	13	1.00 (0.71)	26.62 (13.35)	2,562%	1.00	9.00	2.00	40.00
Niek	6	12	1.33 (0.52)	8.17 (5.87)	514,29%	1.00	9.00	2.00	18.00
Abden	6	12	0.83 (0.41)	10.08 (7.10)	1,114,46%	1.00	11.50	1.00	21.00
Elif	6	12	0.67 (0.52)	20.25 (10.49)	2,922,39%	1.00	21.00	1.00	33.00

N, measurements; M, mean; SD, standard deviation; MBDi, mean baseline difference; Md, median; Max, maximum; A, A phase; and B, B phase.

Further, the overlap measures for the NAP show a medium effect for Lio (82.00; *p* < 0.05), Niek (90.00; *p* < 0.01), and Tila (92.00; *p* < 0.01) and a strong effect for Abden (97.00; *p* < 0.001), Elif (97.00; *p* < 0.001), Nele (99.00; *p* < 0.011), and Kim (100.00; *p* < 0.01). The PAND testifies medium effects for all except Nele and Kim, who show strong effects. A similar picture emerges for the PEM. The Tau-U displays a large change for Lio (0.61; *p* < 0.001), Kim (0.63; *p* < 0.001), Tila (0.69; *p* < 0.01), and Niek (0.69; *p* < 0.001). Abden (0.81; *p* < 0.001), Elif (0.87; *p* < 0.001), and Nele (0.88; *p* < 0.001) show a large to very large change (see Table 9).

**Table 9:** Overlap indices for correctly words read correctly.

Participant	NAP	*p*	PEM	PAND	TauU	*p*
Lio	82.00	<0.05	76.92	70.59	0.61	<0.001
Kim	100.00	<0.01	100.00	100.00	0.63	<0.001
Tila	92.00	<0.01	75.00	82.35	0.69	<0.001
Nele	99.00	<0.001	100.00	94.44	0.88	<0.001
Niek	90.00	<0.01	91.67	75.00	0.69	<0.001
Abden	97.00	<0.001	91.67	83.33	0.81	<0.001
Elif	97.00	<0.001	91.67	86.11	0.87	<0.001

NAP, non-overlapping of all pairs; PEM, percentage of data points exceeding the median; and PAND, the percentage of all non-overlapping data.

The results of the regression analysis at level 2 reveal no statistically significant level (*p* = 0.11) or slope effect (*p* = 0.18) for group 1. Group 2 shows a statistically significant slope from A to B phase (*p* < 0.05) with an increase of 2,503 correct words per intervention session. Group 3 indicates a very similar picture (slope; *B* = 2.502, *p* < 0.05). Overall, a significant slope effect can be observed with a beta coefficient of 1.224 (*p* < 0.05; see Table 10).

**Table 10:** Regression model for words read correctly across all participants (level 2-analysis).

	B	SE	*t*	*p*
*Group 1*				
Intercept	0.000	0.892	0.000	1.00
Trend	0.050	0.323	0.155	0.88
Level	−1.226	0.743	−1.650	0.11
Slope	0.451	0.328	1.378	0.18
*Group 2*				
Intercept	0.050	5.937	0.008	0.99
Trend	0.250	1.183	0.211	0.83
Level	−1.226	0.743	−1.650	0.54
Slope	2.503	1.217	2.057	<0.05
*Group 3*				
Intercept	0.778	2.819	0.276	0.78
Trend	0.250	1.183	0.211	0.93
Level	−1.836	2.012	−0.913	0.54
Slope	2.503	1.217	2.057	<0.05
*Overall*				
Intercept	−1.603	2.911	−0.551	0.58
Trend	0.608	0.558	1.091	0.28
Level	−2.467	1.903	−1.296	0.20
Slope	1.224	0.579	2.114	<0.05

### Social Validity

In terms of social validity, all participants have a very positive attitude towards the intervention overall (see Table 11). With regard to word reading, only Lio and Kim stated “partly agree.” Overall, “completely agree” dominates on all items. The children found that the storytelling helped them, they understood the meaning of the promotion and would like to do more storytelling in school. The students also liked the self-graphing. Only Niek rated “partly agree.”

**Table 11:** Results of social validity questionnaire.

Items	Lio	Kim	Tila	Nele	Niek	Abden	Elif
Storytelling helped me to be able to read words correctly	2	2	3	4	4	4	4
Storytelling helped me learn words and their meanings	3	4	3	4	4	3	4
Storytelling helped me to pronounce sounds correctly	4	4	4	3	4	4	4
I understood well the meaning of the promotion	4	4	4	4	4	3	3
I have learned a lot during storytelling	3	3	4	4	4	4	4
I gladly came to the intervention sessions	4	4	4	4	4	4	4
The self-graphing sheets were fun	3	3	4	4	2	4	4
The stories were great	4	4	3	4	4	4	3
I would like to do more with stories in school	3	4	4	4	4	4	3

0 = completely not agree; 1 = not agree; 2 = partly agree; 3 = agree; and 4 = completely agree.

## Discussion

### Main Findings

The study presented was designed to estimate the effects of a storytelling intervention on the variables: Vocabulary, LSF, and sight word reading in students with German as a second language with and without problem behavior. The background is the increasing number of students with GL2 and at the same time the increase of students with GL2 and weak school performance especially in the area of reading. L2 students are educationally disadvantaged due to their deficits in the language. It is of particular importance to teach these students the language adequately in a motivating way.

Overall, the results are consistent with findings that have looked at multi-component intervention (Foorman and Torgesen, 2001; Donegan and Wanzek, 2021) and the DCT (Paivio, 2008) which states that using verbal and non-verbal system of process information is highly effective in order to finally store information. Moreover, the findings are also consistent with the meta-analysis by Marulis and Neuman (2010) that conveying knowledge explicitly and implicitly in combination leads to the highest effects. Looking at the effectiveness on vocabulary acquisition, it can be seen that all subjects show an immense increase in the B phase, with all baselines being relatively flat. Niek, Kim, and Tila display the weakest effects, although even these can be classified as large. Kim is by far the weakest in the vocabulary pretest with a PR of five. For her, this may be due to the fact that she has great problems building vocabulary overall. In contrast, Tila and Niek perform better in the vocabulary pretest, but unlike Kim, they have greater problems in LRB and the highest problem scores overall in the group. Particularly, problems in attention processing might be a reason here as describe in the literature (Peterson et al., 2013). Abden and Nele are among the strongest performers in terms of vocabulary, but both also show the best results in the vocabulary pretest. It might be easier for them to learn new words if their overall vocabulary is already larger. While Abden has problems with learning-related behavior, which does not seem to play a major role here, Nele shows no problems in this regard. The results of vocabulary acquisition are consistent with the findings of Barwasser et al. (2020, 2021), and Knaak et al. (2021).

Furthermore, for the second dependent variable LSF, the baseline results are higher, i.e., some children have already had experience with German letter sounds, while others reveal a flat baseline with lower values. Niek is the weakest and Lio and Abden the strongest. Niek shows by far the weakest results in the pretest in the area of sound categorization, which could be a reason for his problems in the area of LSF. Overall, Abden is also one of the weakest students in the phonological awareness pretest but sound categorization is his best sub-category with a PR of 28. Like Abden, Lio also has problems in learning behavior which also does not seem to play a major role. However, overall results indicate that the intervention does have a positive impact on LSF which is an important finding since Hulme et al. (2012) has shown that problems in LSF are related to later word-reading difficulties which are referred to almost the same age as the participants of the current study.

With regard to sight word reading, the overall performance is weaker, especially for Lio and Kim. Except for Nele and Elif, the others seem to take longer to automate the words. One explanation for this could be that less-proficient readers often take the non-lexical route because they have greater problems with the lexical route (De Jong et al., 2012). Thus, the children try to decode the words each time instead of storing them as a whole, for which the one second in the measurement is not sufficient. Thus, for these children it takes a longer time until they seem to change the route. Nele and Elif both have much higher scores in phonological awareness and also in pseudoword reading, which should make it easier for them to memorize the words as a whole more quickly, as they are better readers. In reading, they are among the strongest of the subjects in the pretests, along with Abden, who scores third best in overlap-indices. Elif, like Nele, shows no problems in learning-related behavior. Lio and Kim are among the weakest subjects overall in terms of reading and phonological awareness. Perhaps, the Polish L1 also plays a role because L1 background can influence L2 word recognition (Wang and Koda, 2007). According to Catts (1993), phonological awareness is more closely related to word recognition than measures of vocabulary in young first grade children with phonological difficulties and Lio and Kim perform poorly in both areas. Another explanation could be that Lio and Kim might have problems in rapid automatized naming, which is important with regard to naming speed and the retrieval of sight words from the mental lexicon, especially in the German language (Landerl and Wimmer, 2008; Huschka et al., 2021). Nevertheless, Abden, Niek, and Tila also display severe problems in phonological awareness and need longer time to respond to the intervention in word recognition. Niek and Abden have better reading performance in the pretest while Tila performs similarly weak in the pretest as Lio and Kim. So, what could be the reason? In the case of Tila, it could actually be the learning-related behavior that causes problems, or frustration, while in the case of Niek and Abden, the behavioral problems do not seem to have such an impact. One reason could be the overall better reading performance of the two students, which counteracts the problem behavior.

Overall, the intervention seems to work really well for one variable and well for the other two. Storytelling seems to also have an effect on the reading of sight words and goes partly in line by meta-analytic finding by Roberts et al. (2020) who were focused on foundational reading instructions for students with problem behavior in grades K-12 (*g =* 0.86) as well as small group reading instruction for grade 1–4 (e.g., Scammacca et al., 2015). With regard to students who struggled with sight word reading, one can see that even with those displaying slow increase, the improvement seems to come after some time. Another assumption could be that the intervention should have been prolonged in order to achieve greater effects. Also, behavior might have played a role in some cases in combination with very low score in the pretesting. Reflecting on the importance of motivation, especially in language acquisition, self-graphing probably contributed in part to the effects, as studies have pointed to the effectiveness of self-graphing in intervention and especially in reading interventions (Stotz et al., 2008; Bowman-Perrott et al., 2013; Leko, 2016; Guzman et al., 2018; McKenna and Bettini, 2018). Especially regarding the social validity results where all children despite Niek, who seemed to be unsure, rated self-graphing as positive. Moreover, the results of the social validity questionnaire revealed that all participants rated the interventions as positive. With regard to reading, Lia and Kim gave worse scores than the others, but this is also understandable, since both could hardly benefit in sight word reading, also compared to the others.

It is also noticeable that the language background does not necessarily play a role. The Polish background is only noticeable when improving the visual vocabulary, but this does not necessarily mean anything. The sample is too small to be able to make statements about this. Also, problem behavior did not seem to play a role across the board. This may be due to the fact that the children were taken out of the classroom and trained intensively in a small group. In general, small group interventions, especially with regard to reading, have been shown in a meta-analysis by Hall and Burns (2018) to achieve a large effect size for elementary students (*g* = 0.64; also see Nielsen and Friesen, 2012) which can be also referred to Roberts et al. (2020) who examined the effects for primary school students with behavioral problems in a meta-analysis.

### Limitations

In addition to the promising results, there are some imitations: First, the intervention took place during the COVID-19 pandemic, where everyone in the school had to abide by specific rules and it was generally unruly in the school. Groups were therefore not allowed to be mixed from different classes. With regard to reading, it can be seen that those with very weak performance at the phonological level also have greater problems storing the words as sight words. Here, it would probably make sense to stay one level lower and train the LSF and other aspects of decoding more intensively. Furthermore, this is a multiple baseline study, which means that we focused on individual students, making it difficult to generalize the results. Nevertheless, the results give important indications with regard to the support of struggling students with GL2 with and without behavioral difficulties. The advantage of a multiple baseline study is that it allows us to see individual learning trajectories and to find out specifically how the intervention is received by different students.

Another limitation is that there is a certain probability that the children have also become better through the repeated measurements each time after the sessions. We have tried to counteract this by randomizing the order of the items in each test, but we cannot exclude it for sure. However, since there are no trends in the baselines where only testing was done, it could be argued that the influence of testing was not too great. A further minor limitation is the measurement time point of the first group in the baseline, since across the board at least five measurement time points are always recommended in each phase. After Kratochwill et al. (2013), however, at least three measurement time points are also sufficient to be able to make a statement. Due to time constraints, it was not possible to extend the baseline. And, as with all multi-component interventions, of course, one does not know which component worked for which parts. At the current time, it is not possible to say exactly to which parts the various components (such as self-graphing and implicit vs. explicit teaching) have had on the dependent variables. Since this intervention seems to work in this package, it is basically not the intention to examine the individual parts separately, as the package is very easy and straightforward to implement in the classroom.

### Implications

A first goal would be to estimate the storytelling intervention on a larger sample and make generalized statements. Furthermore, the intervention would be compared to other interventions in order to see which support option seem to be most effective in the area of language acquisition. In the course of this, one could also look at whether the method also works with a whole class or if it is limited to small groups. In the context of digitalization and especially the current school closures worldwide due to the COVID-19 pandemic, which has once again shown how important digital learning is in schools, the storytelling intervention could be digitalized and made available *via* apps or web-based tools.

The intervention in its current form was rated very positively, which gives us an indication that despite the overall good effects on all three dependent variables, the intervention is accepted across all participants. The further implication of this is to continue to conduct the social validity survey in future research to gain more insight into the overall acceptability of the intervention, which according to Briesch et al. (2013) is a necessity in intervention research. Last implications are the different languages and behavioral problems. It would be interesting to see whether the effects differ between children from different language backgrounds (Wang and Koda, 2007). In addition, one could also record the abilities in the surveyed variables in the L1 in order to identify possible correlations here. Furthermore, the study looked at children with learning-related behavior problems. A continuation would be to see if the intervention would also help with students with disruptive behavior, which is a big challenge for teachers today (Rosenberg and Jackman, 2003). Also, measuring rapid automatized naming beforehand would be interesting since it is linked to rapid word retrieval and reading, particularly in the German language which is more transparent than, e.g., English (Landerl and Wimmer, 2008).

## Conclusion

It is enormously important to support struggling language learners in all components of a language in order to provide equal chances with respect to school and later job possibilities, especially to actively address the results of the PISA survey (OECD, 2019). Also, Morgan et al. (2015) showed that first graders with reading problems are more likely to show off task-behavior and general problem behavior in grade 3. Also considering the meta-analysis by Chow and Wehby (2018) on the negative relationship of language problems and behavioral difficulties, it is imperative to counteract this, particularly when students already display some kind of problem behavior. Also, one should consider the Matthew effect that stronger readers become stronger and weaker readers become weaker particularly in the first years of school because they start to dislike reading (Stanovich, 1986). Thus, early prevention in school failure is really important, specifically for students with GL2 and those with additional problem behavior who struggle with reading. This storytelling approach should give teachers, educators, and researchers an indication of how an intervention in this area could look like which can train different areas of language at the same time and matches the concept of inclusion by Booth and Ainscow (2011) to integrate students with different competencies and characteristics as well as from different backgrounds.

## Data Availability Statement

The raw data supporting the conclusions of this article will be made available by the authors, without undue reservation.

## Ethics Statement

Ethical review and approval was not required for the study on human participants in accordance with the local legislation and institutional requirements. Written informed consent to participate in this study was provided by the participants’ legal guardian/next of kin.

## Author Contributions

AB developed the idea (conceptualization, material, and methodology), conducted the study and analyzed the data. AB, JB, and MG did the interpretation of data. All authors read and approved the final manuscript.

### Conflict of Interest

The authors declare that the research was conducted in the absence of any commercial or financial relationships that could be construed as a potential conflict of interest.
